# Six-Year Follow-Up Outcomes of Catheter Ablation of Para-Hisian Accessory Pathways

**DOI:** 10.3389/fcvm.2021.692945

**Published:** 2021-09-07

**Authors:** Qingxing Chen, Lili Xu, Tian Zou, Kuang Cheng, Yunlong Ling, Ye Xu, Yang Pang, Guijian Liu, Wenqing Zhu, Junbo Ge

**Affiliations:** Department of Cardiology, Zhongshan Hospital, Fudan University, Shanghai Institute of Cardiovascular Diseases, Shanghai, China

**Keywords:** para-hisian accessory pathway, catheter ablation, recurrence, follow up, ablation time

## Abstract

**Background:** Ablation of para-hisian accessory pathways (APs) remains challenging due to anatomic characteristics, and a few studies have focused on the causes for recurrence of radiofrequency ablation of para-hisian APs.

**Objective:** This retrospective single center study aimed to explore the risk factors for recurrence of para-hisian APs.

**Methods:** One hundred thirteen patients who had para-hisian AP with an acute success were enrolled in the study. In the 6-year follow-up, 15 cases had a recurrent para-hisian AP. Therefore, 98 patients were classified into the success group, while 15 patients were classified into the recurrence group. Demographic and ablation characteristics were analyzed.

**Results:** Gender difference was similar in two groups. The median age was 36.2 years old and was younger in the recurrence group. Maximum ablation power was significantly higher in the success group (29 ± 7.5 vs. 22.9 ± 7.8, *p* < 0.01). Ablation time of final target sites was found to be markedly higher in the success group (123.4 ± 53.1 vs. 86.7 ± 58.3, *p* < 0.05). Ablation time <60 s was detected in 12 (12.2%) cases in the success group and 7 (46.7%) cases in the recurrence group (*p* < 0.01). Occurrence of junctional rhythm was significantly higher in the recurrence group (25.5% vs. 53.3%, *p* < 0.05). No severe conduction block, no pacemaker implantation, and no stroke were reported. Junctional rhythm during ablation (OR = 3.833, 95% CI 1.083–13.572, *p* = 0.037) and ablation time <60 s (OR = 5.487, 95% CI 1.411–21.340, *p* = 0.014) were independent risk factors for the recurrence of para-hisian AP.

**Conclusions:** With careful and accurate mapping, it is relatively safe to ablate para-hisian AP. If possible, proper extension of ablation time could reduce the recurrence rate of para-hisian APs.

## Introduction

Radiofrequency (RF) catheter ablation is well established as a definitive therapy for atrioventricular accessory pathways (AP) (1). In experienced centers, success rate of AP ablation has exceeded 95% with a low rate of complications (2). However, ablation of APs located in para-hisian region remains a challenging task due to anatomic proximity to normal conduction system. The para-hisian regions often refer to the superoparaseptal and midseptal. APs are classified as superoparaseptal if the AP potential and His potential are simultaneously recorded from the diagnostic catheter placed at the His bundle region. APs are classified as midseptal if ablation is achieved through the mapping-ablation catheter located in an area bounded superiorly by the electrode recording the His potential, and posteroinferiorly by the coronary sinus, as marked by the vortex of curvature in the coronary sinus catheter. According to the published data, among accessory AV pathways, those located in the anteroseptal space are the least. The prevalence of the anteroseptal space, mainly on the right side of the heart, has been reported to be 2–10% (3). Recurrence rate and risk of atrioventricular (AV) block are relatively higher in procedures of eliminating para-hisian APs (3–5), but little research has carefully studied the causes for recurrence of radiofrequency ablation of para-hisian APs. This retrospective study aimed at exploring the risk factors for recurrent para-hisian APs.

## Method

### Patient Selection

A total of 13,952 consecutive patients diagnosed as supraventricular tachycardia (SVT) or ventricular pre-excitation underwent radiofrequency ablation in our laboratory from January 2009 to December 2019. One hundred thirteen patients who had an AP located at the para-hisian region and with an acute success ablation were enrolled in the present study. Approval for this retrospective study was obtained from the Committee on Clinical Investigation at Zhongshan Hospital Affiliated to Fudan University.

### Electrophysiological Study

All anti-arrhythmic therapies were discontinued for five half-lives before the study. Written informed consent was obtained from all the patients before the procedure. Under fluoroscopic visualization, two or three quadripolar catheters were advanced to the high atrial atrium, His bundle, and/or right ventricular apex. A decapolar catheter was placed in the coronary sinus. The most proximal bipole of this catheter was positioned at the coronary sinus ostium. Intra-cardiac electrograms were simultaneously displayed with electrocardiographic leads I, aVF, and V1 on a multi-channel recording system at a paper speed of 150–200 mm/s (EP MED Systems). The bipolar signals were filtered at 30–500 Hz. We induced tachycardia by programmed electrical stimulation. Following documenting narrow QRS tachycardias, differential diagnosis was performed by atrial or ventricular extra-stimulations, atrial or ventricular entrainment pacing, or para-hisian pacing. Conduction intervals and refractory periods were measured and defined as previously described (6). The diagnosis of various forms of atrioventricular reentrant tachycardias (AVRTs) incorporating APs was made and defined according to the previously described criteria (1). Localization and identification of the APs were achieved by careful mapping of the atrial or ventricular activation pattern, or both, using unipolar and bipolar electrograms recorded from regular electrode catheters as well as a steerable ablation catheter. The insertion sites of APs were considered to be para-hisian when a discernible His bundle potential was recorded (at the site of earliest atrial activation) during AVRT or following ablation of a manifest AP with disappearance of ventricular pre-excitation, or the successful ablation target located within 5 mm in distance (measured from the X-ray image) to the His electrode, which manifest significant His bundle potential for concealed AP and intermittent pre-excited AP or after successful elimination of a manifest AP. The possibility of atrioventricular node reentry tachycardia (AVNRT) was excluded by noting earlier atrial excitation without changing the activation sequence by delivery of a ventricular premature beat during tachycardia at a time when the His bundle was refractory. The possibility that a reciprocating tachycardia might incorporate a retrogradely conducting concealed nodo-ventricular pathway was evaluated by isolating the atria from the tachycardia circuit by delivery of an atrial or ventricular extra-stimulus during tachycardia.

### Mapping and Catheter Ablation

A 7 Fr quadripolar deflectable catheter with a 4-mm distal electrode and a 2-mm inter-electrode distance between the distal two electrodes (Mansfield–Webster) was introduced percutaneously into a femoral vein and advanced to the right atrium. Using the His bundle and the coronary sinus catheters for reference, we positioned the tip of the electrode catheter at various sites in the AV junctional area to map the para-hisian APs. Mapping of the accessory pathway was performed during sinus rhythm, steady-rate ventricular pacing, or SVT as determined by pathway conduction characteristics. The earliest atrial or ventricular activation was assessed on both bipolar and unipolar signals, and signal morphology was assessed, looking for high-frequency signals consistent with an accessory pathway potential. All initial mapping procedures were performed from an inferior approach with an RF ablation catheter. Visualization during mapping was performed with fluoroscopy and three-dimensional electroanatomic mapping (CARTO System, Biosense Webster) in each patient. RF ablation would be performed in the para-hisian APs when the effective refractory period of AP is <250 ms, or the RR intervals during induced pre-excited atrial fibrillation is <220 ms. When the presence of the para-hisian APs were confirmed, we had three different ablation approaches which targeted at the right atrial anteroseptal (RA) as the initial target, right ventricular septum (RV) by catheter inversion technique as the second, and the non-coronary cusp (NCC) as the last target. Aortic angiography was performed to establish the location of coronary arteries and to delineate the anatomical features of the coronary cusps before the NCC was mapped. Before the RF energy was delivered, disappearance of delta wave on surface ECG or ventriculo-atrial conduction block could sometimes be detected by catheter compression. This phenomenon is called success by catheter compression. The delivery of RF energy was carefully targeted to the region with earliest retrograde atrial activation via the AP or earliest antegrade ventricle activation during pre-excitation. RF power delivery was applied by titration method with a temperature control mode (temperature limit: 55–60 °C), and the max power delivered was limited to 30–40 W. The ablation procedure was discontinued immediately if the rise of impedance, AV block, chest pain, hypotension, or severe bradycardia were noted. A power of 5–10 W for 20 s was delivered initially. Anterograde and retrograde conduction properties were evaluated immediately after each current application. If it was effective and safe, time to success was recorded, then energy gradually increased to max energy and was continued for a range of 20–60 s (repeat two to four times) depending on the operator. If it did not work and without heart block for a certain current delivered at least 20 s, a more aggressive power (5–10 W or more) was delivered for at least 20 s until it was effective. If it failed to abolish the APs after at least twice by the maximal ablation current or manifest rapid junctional reaction or transient AV block or bundle block, then we shift to the other ablation strategy (from RA to RV and NCC in sequence). Ablation was considered acute success if there was no evidence of residual preexcitation or abnormal retrograde pathway conduction based on decremental retrograde conduction and adenosine administration for 15–30 min after the successful ablation lesion.

### Patient Follow-Up

The patients were followed up at 1-month, 3-month, 6-month, 1-year, and then every year after the operations. Recorded data included symptoms, 12-lead ECG or Holter recordings, and other complications. Recurrences were evident with either a return in pre-excitation on the 12-lead ECG and/or documented SVT. Procedural complications were tracked for any significant mortality or morbidity, especially changes in AV conduction.

### Statistical Analysis

Continuous variables were expressed as the means and standard deviations. Categorical variables were summarized as the counts and percentages in each category. For comparison of variables, Student's *t*-test and Pearson χ2 test were used. Multi-factor logistic regression model was used to analyze the related factors of recurrences. A *p* < 0.05 was considered significant.

## Results

**Figure 1:**
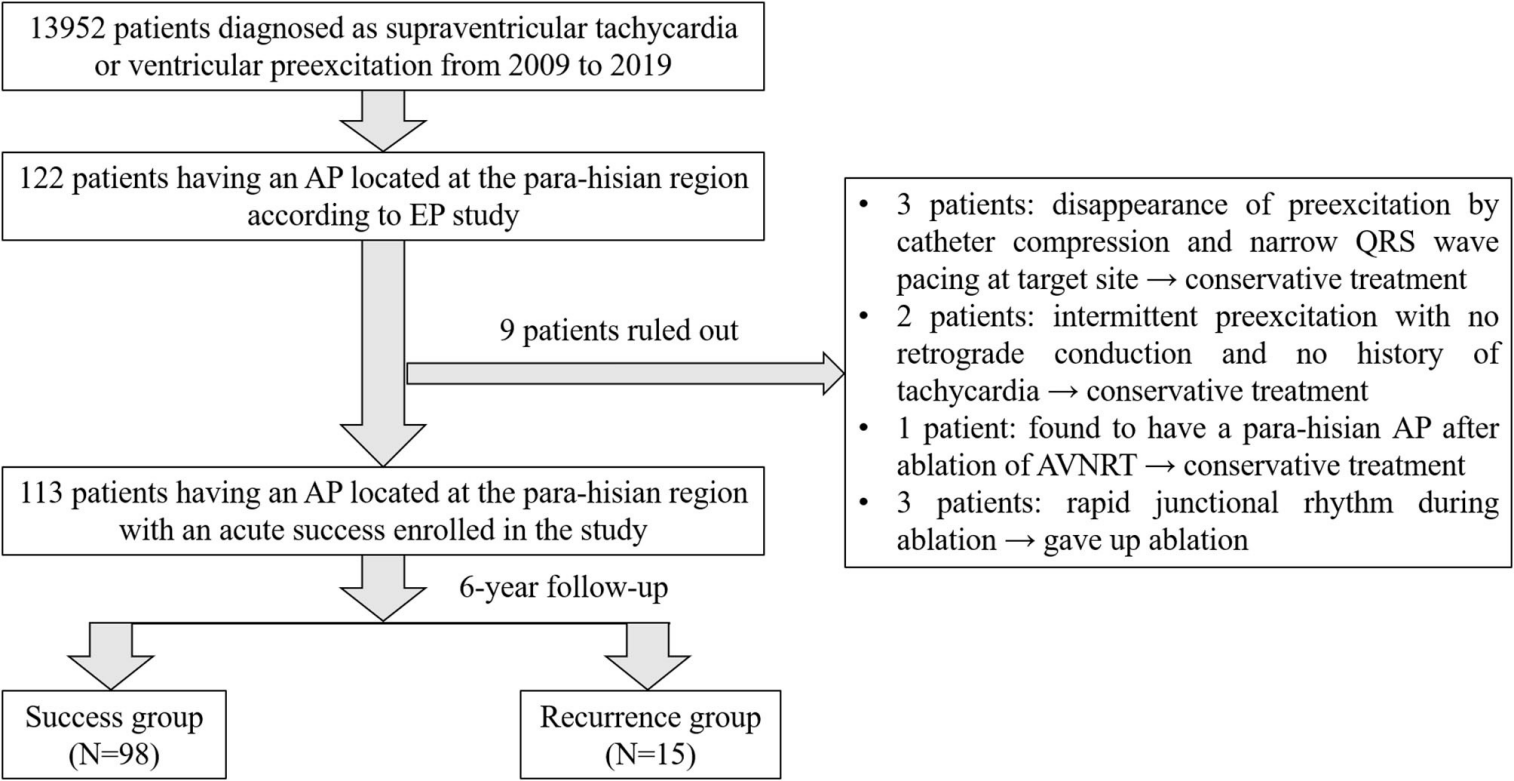
Flowchart of patient enrollment.

A total of 13,952 consecutive patients were diagnosed with SVT or ventricular pre-excitation, and underwent RF ablation in our laboratory from January 2009 to December 2019. Among them 122 patients were considered to have an AP located at the para-hisian region according to the EP study. Disappearance of preexcitation by catheter compression and narrow QRS wave by pacing at a target site were observed in three patients who chose conservative treatment. Two patients had intermittent preexcitation with no retrograde conduction and no history of tachycardia and chose conservative treatment as well. One patient was found to have a para-hisian AP after ablation of AVNRT and then chose conservative therapy. Three patients with rapid junctional rhythm (fast junctional tachycardia, TCL <350 ms) during ablation were unwilling to accept the risk of pacemaker and gave up the ablation. Finally, 113 patients who had an AP located at the para-hisian region with an acute success were enrolled in the present study. In the 11-year follow-up, 15 cases had a recurrent para-hisian AP. Therefore, 98 patients were classified into the success group, while 15 patients were classified into the recurrence group (see Figure 1).

### Demographic and Clinical Characteristics

Gender was similar in success and recurrence group. The median age was 36.2 years old and was younger in recurrence group, but the average age was similar in both groups. One hundred five patients had a history of SVT, including 90 (91.8%) in the success group and 15 (100%) in the recurrence group. The onset age of SVT was a bit younger in the recurrence group, but no significant difference was observed in both groups. Sixty patients had a manifest pre-excitation, including one type A pre-excitation (with multiple APs in success group) and 59 type B pre-excitation. Nine patients had a previous history of atrial fibrillation. Ten patients had a history of wide QRS tachycardia, including three with antidromic AVRT and seven with orthodromic AVRT and right bundle branch block. Twenty patients had repeat EP study and ablation, including 18 (18.4%) in the success group and two (13.3%) in the recurrence group. These 18 patients having repeat EP study in the success group failed to ablate para-hisian APs in other centers and then transferred to our center. Two cases in the success group underwent ablation for the third time. Demographic and clinical features are summarized in Table 1.

**Table 1:** Patient demographic characteristics.

	**Total**	**Success**	**Recurrence**	***p*-Value**
	**(*N* = 113)**	**(*N* = 98)**	**(*N* = 15)**	
Male	76 (67.3%)	66 (67.3%)	10 (66.7%)	0.58
Age (years old)	36.2 ± 14.8	37.2 ± 14.6	29 ± 14.7	0.08
Palpitation	106 (93.9%)	91 (92.9%)	15 (100%)	0.59
SVT	105 (92.9%)	90 (91.8%)	15 (100%)	0.59
Onset age of SVT (years old)	27.9 ± 14.5	28.8 ± 14.4	21.7 ± 14.2	0.08
Pre-excitation	60 (53.1%)	49 (50%)	11 (73.3%)	0.1
Intermittent WPW	8 (13.3%)	5 (5.1%)	3 (20%)	0.07
Af history	9 (8%)	7 (7.1%)	2 (13.3%)	0.34
Wide QRS tachycardia	10 (8.8%)	8 (8.2%)	2 (13.3%)	0.62
Previous EP study				
Primary	93 (82.3%)	80 (71.6%)	13 (86.7%)	0.62
Repeat	20 (17.7%)	18 (18.4%)	2 (13.3%)	0.62
Echocardiographic abnormality	27 (23.8%)	22 (22.4%)	5 (33.3%)	0.35
Left atrial enlargement	12 (10.6%)	10 (10.2%)	2 (13.3%)	0.66
Hypertrophic cardiomyopathy	2 (1.8%)	2 (2.0%)	0 (0%)	0.93
LV systolic dysfunction	4 (3.5%)	3 (3.1%)	1 (6.7%)	0.44
Heart failure	3 (2.7%)	2 (2%)	1 (6.7%)	0.35
Congenital heart disease	3 (2.7%)	2 (2%)	1 (6.7%)	0.35
Tricuspid regurgitation	5 (4.4%)	3 (3.1%)	2 (13.3%)	0.13
Ebstein's anomaly	2 (1.8%)	1 (1%)	1 (6.7%)	0.25
Permanent left superior vena cava	2 (1.8%)	2 (2%)	0 (0%)	0.58
Atresia of coronary sinus	1 (0.9%)	1 (1%)	0 (0%)	0.85

*SVT, supraventricular tachycardia; EP, electrophysiological*.

### Echocardiographic Findings

We performed echocardiography in all enrolled patients. Occurrence of echocardiographic abnormality was observed in 27 patients, including 22 (22.4%) in the success group and five (33.3%) in the recurrence group. No difference was found between the two groups. Left atrial enlargement was found in 12 cases (10 success cases and two recurrent cases), and hypertrophic cardiomyopathy was found in two cases (both success cases). Four cases presented with left ventricular systolic dysfunction. Three cases were found to have congenital heart disease, including two cases with atrial septal defect and one case with patent ductus arteriosus. Two patients were found to have Ebstein's anomaly with moderate to severe tricuspid regurgitation. One case in the success group was found to have atresia of the coronary sinus and permanent left superior vena cava. Echocardiographic features are summarized in Table 1.

### Electrophysiological Study and Ablation

AVRTs were induced during operation in 100 cases, including 87 (88.9%) cases in the success group and 31 (86.7%) cases in the recurrence group. Four inducing methods were applied, including programmed atrial stimulation, programmed ventricular stimulation, bilateral stimulation, and stimulation with isoprenaline induction. No difference was found in the AP conduction ratio between the success and recurrence groups. Bidirectional AP conduction was observed in 56 cases, including 45 (45.9%) cases in the success group and 11 (73.3%) cases in the recurrence group. Retrograde AP conduction was detected in 54 patients, while antegrade AP conduction was detected in three patients. Ten cases presented with multiple APs, including three cases with right AP and seven cases with left AP. One case was detected with triple APs. We tried multiple target ablation in 17 patients in the success group and three patients in the recurrence group. Final target sites of ablation consisted of RA (96 cases, including six cases failed to ablate at other sites and then back to RA), RV (11 cases), and NCC (3 cases). Disappearance of pre-excitation was detected by catheter compression in 19 cases. Maximum ablation power was significantly higher in the success group (29 ± 7.5 vs. 22.9 ± 7.8, *p* < 0.01). Ablation time of the final target sites was found to be markedly higher in the success group (123.4 ± 53.1 vs. 86.7 ± 58.3, *p* < 0.05). Ablation time <60 s was detected in 12 (12.2%) cases in the success group and 7 (46.7%) cases in the recurrence group (*p* < 0.01), indicating the prominent role of ablation time in the success of operation. Eleven cases in the success group and six cases in the recurrence group presented with rapid junctional reaction during operation. Occurrence of junctional rhythm was significantly higher in the recurrence group (*p* < 0.05). Fourteen cases in the success group and two cases in the recurrence group presented with slow junctional rhythm, and no significant difference was identified between the two groups. No difference of atrioventricular delay or block was found in both groups. We applied dexamethasone during operation in four patients in the success group who experienced transient AVD or AVB to reduce local edema and prevent further conduction abnormality. Details about the EP study and ablation are shown in Table 2.

**Table 2:** EP study and ablation.

	**Total**	**Success**	**Recurrence**	***p*-Value**
	**(*N* = 113)**	**(*N* = 98)**	**(*N* = 15)**	
AVRT induced during operation	100 (88.5%)	87 (88.8%)	13 (86.7%)	0.68
Inducing method				
Atrial	26 (23%)	22 (22.4%)	4 (23.7%)	0.51
Ventricular	35 (31%)	33 (33.6%)	2 (13.3%)	0.46
Both	39 (34.5%)	32 (32.7%)	7 (46.7%)	0.74
Isoprenaline	22 (19.5%)	21 (21.4%)	1 (6.7%)	0.3
Endless tachycardia	3 (2.7%)	2 (2%)	1 (6.7%)	0.35
Af during ablation	8 (7.1%)	7 (7.1%)	1 (6.7%)	0.71
AP conduction				
Bidirectional	56 (49.6%)	45 (45.9%)	11 (73.3%)	0.06
Retrograde only	54 (47.8%)	50 (51%)	4 (23.7%)	0.1
Antegrade only	3 (2.65%)	3 (3.1%)	0 (0%)	0.66
Multiple Aps	10 (9%)	9 (9.2%)	1 (6.7%)	0.74
Multiple target ablation	20 (16.8%)	17 (17.3%)	3 (20%)	0.72
Final target				
RA	96 (85%)	85 (86.7%)	13 (86.6%)	0.95
RV	11 (11%)	10 (10.2%)	2(13.4%)	0.9
NCC	3 (2.7%)	3 (3.1%)	0 (0%)	0.65
Success by catheter compression	19 (16.8%)	15 (15.3%)	4 (26.7%)	0.27
Maximum power (W)	**28.2 ± 7.8**	**29 ± 7.5**	**22.9 ± 7.8**	**0.004**
Time to success (s)	5.7 ± 5	6 ± 5	4 ± 5	0.16
Ablation time of final target (s)	**118 ± 54.6**	**123.4 ± 53.1**	**86.7 ± 58.3**	**0.04**
Ablation time <60 s	**19 (16.8%)**	**12 (12.2%)**	**7 (46.7%)**	**0.003**
Reaction during RFCA				
Junctional rhythm	**33 (29.2%)**	**25 (25.5%)**	**8 (53.3%)**	**0.03**
AVD	4 (3.5%)	3 (3.1%)	1 (6.7%)	0.44
AVB	3 (2.7%)	2 (2%)	1 (6.7%)	0.35
Dexamethasone application	4 (3.5%)	4 (4.1%)	0 (0%)	0.56

*AVRT, atrioventricular reentrant tachycardia; AP, accessory pathway; RA, right atrial anteroseptal; RV, right ventricular septum; NCC, non-coronary cusp*.

*Risk factors with a significant difference (p < 0.05) are in bold*.

### Follow-Up

The time span of follow-up lasted for 11 years, and the average time of follow-up was 6.1 ± 5.7 years. In the follow-up of 113 cases with an acute success, 15 cases had a recurrent para-hisian AP. The average time of recurrence was 4.8 ± 9.1 months. The 1-month recurrence rate was 6.29% (seven cases), while the 3-month recurrence rate was 9.73% (11 cases) and the 6-month recurrence rate was 11.5% (13 cases). The recurrence of another two cases occurred in 11 and 36 months post operation, respectively. Four cases had a recurrent preexcitation with no history of tachycardia, while the other 11 cases had a history of tachycardia, including one patient with aggravating symptoms, five patients with alleviating symptoms, and five patients with similar symptoms compared with manifestations before operation. For the six patients with aggravating or similar symptoms, two chose anti-arrhythmic drugs, and the other four chose ablation again (two in our center and two in other centers). New onset of complete right bundle branch block was detected in only one case. No severe conduction block, no pacemaker implantation, and no stroke were reported in the 11-year follow-up.

### Factors Predicting the Recurrence of Para-Hisian Accessory Pathway

Multifactor logistic regression analysis showed that junctional rhythm during ablation (OR = 3.833, 95% CI 1.083–13.572, *p* = 0.037) and ablation time <60 s (OR = 5.487, 95% CI 1.411–21.340, *p* = 0.014) were independent risk factors of the recurrence of para-hisian AP (Table 3).

**Table 3:** Multi-factor logistic regression analysis of factors predicting the recurrence.

	**OR**	**95% CI**	***p*-Value**
Junctional rhythm during ablation	**3.833**	**1.083–13.572**	**0.037**
Ablation time <60 s	**5.487**	**1.411–21.340**	**0.014**
Maximum ablation energy	0.939	0.866–1.018	0.126

*Risk factors with a significant difference (p < 0.05) are in bold*.

## Discussion

The ablation of para-hisian APs remains a challenging task due to anatomic characteristics. Few researchers have focused on the causes for recurrence of radiofrequency ablation of para-hisian APs. This retrospective study was a relatively large sample size, long-term follow-up, single center study, aiming at exploring the risk factors for recurrent para-hisian APs. Our data reported that patients with a para-hisian AP mainly consisted of young and middle-aged adults with a median age of 36.2 years old, consistent with previous studies (5, 7). The average age was even younger in the recurrence group, which is associated with a probably higher risk of junctional rhythm and more conservative manipulation during ablation.

Some small-sample-size studies in the early years reported that severe atrioventricular conduction disturbance occurred in about 0–4% cases, even leading to pacemaker implantation (3–5, 7, 8). Kovach et al. reported in an ablation study in children that three patients (1.2%) had significant complications during the study period while two patients had Mobitz II second-degree AV block immediately after ablation (9). In our study, despite the occurrence of atrioventricular delay or block during operation, neither severe conduction block nor pacemaker implantation were reported in the 6-year follow-up, indicating that with careful and accurate mapping, it is relatively safe to ablate para-hisian APs. On one hand, the His bundle carries a sleeve of fibrous annular tissue for variable distances into the ventricle, losing this insulation as the right bundle exits to the myocardium. On the other hand, anatomically, the His bundle is a ventricular and anterior structure, whereas the compact AV node is a midseptal and atrial structure. Therefore, it is safer to ablate the anteroseptal region (10).

Commonly used ablation energy for para-hisian pathway was applied by titration method according to published studies. Some researchers suggested that after successful ablation of an accessory pathway, one additional “safety” application was given after 1 min to the same site to minimize the possibility of a late recurrence of accessory pathway conduction (5). On the other hand, some researchers held the view that repeat radiofrequency pulses (“safety pulses”) after effective pulses did not predict resumption of accessory pathway conduction (11). A few studies have focused on the maximum ablation power and ablation time previously, and the relationship with the recurrence of the ablation of para-hisian pathway. Our data showed that ablation time of final target sites was significantly lower in the recurrence group, and ablation time <60 s was an independent risk factor for the recurrence, indicating the prominent role of ablation time in the long-term success of operation. In addition, we found that occurrence of junctional rhythm was significantly higher in the recurrence group and that junctional rhythm during operation was an independent risk factor for the recurrence. We considered that this might be associated with conservative manipulation due to junctional rhythm during operation and subsequent insufficient ablation time and power.

The overall acute procedural success rate in our study is 97.4%, higher than some data reported (9, 11). Moreover, the overall long-term rate of freedom from recurrence in this study is 86.7%. In patients with previously failed ablation, careful mapping of a para-hisian AP at the NCC has been suggested as an alternative approach (12, 13), which is consistent with our data. We tried multiple target ablation in 20 patients. Three cases with failed ablation at other sites achieved success at NCC. However, no difference was found between success and recurrence group at different target sites of ablation. Studies have shown that the success rate of ablation was significantly higher at the right ventricular or NCC, compared with the right atrium (14). It is worth mentioning that six cases in our study achieved success finally at the right atrium after multiple (including NCC) mapping and ablation. It is reported that concealed accessory pathway, multiple APs, and structural heart disease were associated with the recurrence (11, 15, 16). However, no relationship was detected in this study between the recurrence rate and concealed accessory pathway, multiple APs, and structural heart disease (including congenital heart disease, Ebstein's anomaly, hypertrophic cardiomyopathy, etc.).

## Limitations

The major limitation of our study is that this was not a prospective, randomized study comparing different ablation energy and time. In addition, contact force catheter were not applied in this retrospective study, so other relevant factors such as the contact force and catheter stability were not recorded. The relationship between junctional rhythm during ablation, ablation time <60 s, and recurrence of catheter ablation of para-hisian accessory pathways need a larger sample investigation in the future.

## Conclusions

Junctional rhythm during ablation and ablation time <60 s were independent risk factors for the recurrence of para-hisian AP. With careful and accurate mapping, it is relatively safe to ablate para-hisian APs. If possible, proper extension of ablation time could reduce the recurrence rate of para-hisian APs.

## Data Availability Statement

The raw data supporting the conclusions of this article will be made available by the authors, without undue reservation.

## Ethics Statement

The studies involving human participants were reviewed and approved by the Committee on Clinical Investigation at Zhongshan Hospital Affiliated to Fudan University. The patients/participants provided their written informed consent to participate in this study.

## Author Contributions

QC contributes to the whole design of the study. QC, LX, and TZ contribute to data analysis and manuscript preparation. KC, YL, YX, YP, and GL contributes to the operations and enrolment of the participants. JG and WZ provide suggestions in the study process. All authors contributed to the article and approved the submitted version.

## Funding

This work was supported by Shanghai Science and Technology Commission (17DZ1930303), Shanghai Municipal Commission of Economy and Informatization (GYQJ-2018-2-05), Scientific Research and Development Fund of Zhongshan Hospital, Fudan University (No: 331), and National Natural Science Foundation of China (82100466).

## Conflict of Interest

The authors declare that the research was conducted in the absence of any commercial or financial relationships that could be construed as a potential conflict of interest.

## Publisher's Note

All claims expressed in this article are solely those of the authors and do not necessarily represent those of their affiliated organizations, or those of the publisher, the editors and the reviewers. Any product that may be evaluated in this article, or claim that may be made by its manufacturer, is not guaranteed or endorsed by the publisher.

## Abbreviations

RF: radiofrequency
AP: accessory pathways
AV: atrioventricular
SVT: supraventricular tachycardia
EP: electrophysiological
AVRT: atrioventricular reentrant tachycardia
AVNRT: atrioventricular node reentry tachycardia
RA: right atrial
RV: right ventricular
NCC: non-coronary cusp.
